# Identifying strategies for implementing a clinical guideline for cancer-related fatigue: a qualitative study

**DOI:** 10.1186/s12913-023-09377-9

**Published:** 2023-04-24

**Authors:** Elizabeth J. Pearson, Linda Denehy, Lara Edbrooke

**Affiliations:** 1 Health Services Research and Implementation Science, Peter MacCallum Cancer Centre, Melbourne, Australia; 2 Sir Peter MacCallum Department of Oncology, The University of Melbourne, Melbourne, Australia; 3 Department of Physiotherapy, The University of Melbourne, Melbourne, Australia

**Keywords:** Barriers, Facilitators, Consumer, Health professional, Implementation, Fatigue, Guideline, Education, CFIR

## Abstract

**Background:**

Clinical practice guidelines assist health professionals’ (HPs) decisions. Costly to develop, many guidelines are not implemented in clinical settings. This paper describes an evaluation of contextual factors to inform clinical guideline implementation strategies for the common and distressing problem of cancer-related fatigue (CRF) at an Australian cancer hospital.

**Methods:**

A qualitative inquiry involving interviews and focus groups with consumers and multidisciplinary HPs explored key Canadian CRF guideline recommendations. Four HP focus groups examined the feasibility of a specific recommendation, while a consumer focus group examined experiences and preferences for managing CRF. Audio recordings were analysed using a rapid method of content analysis designed to accelerate implementation research. Strategies for implementation were guided by the Consolidated Framework for Implementation Research.

**Results:**

Five consumers and 31 multidisciplinary HPs participated in eight interviews and five focus groups. Key HP barriers to fatigue management were insufficient knowledge and time; and lack of accessible screening and management tools or referral pathways. Consumer barriers included priority for cancer control during short health consultations, limited stamina for extended or extra visits addressing fatigue, and HP attitudes towards fatigue. Enablers of optimal fatigue management were alignment with existing healthcare practices, increased HP knowledge of CRF guidelines and tools, and improved referral pathways. Consumers valued their HPs addressing fatigue as part of treatment, with a personal fatigue prevention or management plan including self-monitoring. Consumers preferred fatigue management outside clinic appointments and use of telehealth consultations.

**Conclusions:**

Strategies that reduce barriers and leverage enablers to guideline use should be trialled. Approaches should include (1) accessible knowledge and practice resources for busy HPs, (2) time efficient processes for patients and their HPs and (3) alignment of processes with existing practice. Funding for cancer care must enable best practice supportive care.

**Supplementary Information:**

The online version contains supplementary material available at 10.1186/s12913-023-09377-9.

## Introduction / Aim

Clinical practice guidelines are designed to assist health care professionals (HPs) to provide the most appropriate care for their patients [1]. Using best available evidence, guideline development is slow and expensive [2]. Yet, many guidelines are not implemented [3], with potential benefits for patients lost. Reasons why effective interventions recommended in guidelines are not used include lack of leadership on treatment policy, lack of awareness of or trust in the guideline and complexity of guideline recommendations [4]. The feasibility of guideline use in real clinical settings has rarely been explored in any condition. Guidelines often include several recommendations that were developed by stakeholders (including content experts, health professionals, consumers) based on evidence, but their practicability is not commonly tested [5]. Strategic approaches are needed to overcome barriers and facilitate enablers to guideline use [6, 7]. According to the Consolidated Framework for Implementation Research (CFIR), characteristics of the guideline (intervention), health practitioners, organisations (internal setting), external context (e.g. consumer needs and policy) and implementation strategies should all be considered [8].

Fatigue is recognised as a common, distressing symptom during and after cancer treatment [9, 10]. Approximately 50% of patients during treatment, and 30% of survivors after treatment experience prolonged and debilitating fatigue [11]. Persistent fatigue at moderate to severe levels is associated with disability, psychological disorders [12, 13] and poor performance status [11, 14]. International guidelines for cancer-related fatigue (CRF) recommend systematic screening for fatigue, assessment of contributing factors, and interventions including symptom management, exercise, fatigue education and sleep enhancement [15]. Many Australian cancer care professionals do not routinely use CRF guidelines [9, 16], and the feasibility of their use is not known. A study of clinician and consumer perceptions about applying a Canadian CRF guideline [13] highlighted insufficient detail to perform guideline recommendations, and a lack of clinical tools including assessments and consumer education [9]. A Canadian study reported lack of HP knowledge, resources and system barriers, together with inconsistent provider-patient communication of fatigue accounted for their CRF practice gap [17]. These and other barriers need to be addressed to enable CRF guideline use and optimal care for cancer survivors [15]. Currently, the best approaches to implementing CRF guidelines in complex “real-world” cancer practices are unknown [15], with scant data related to patient outcomes.

This paper describes the development of strategies for implementing a clinical guideline for CRF at an Australian specialist cancer hospital, using the lens of the CFIR [8]. The research question was ‘*What strategies could increase the feasibility of CRF guideline use?’* Study aims were (1) to assess barriers and enablers to implementing a fatigue guideline at a Comprehensive Cancer Centre, and (2) to identify strategies to support guideline implementation at the Centre.

## Methods

This qualitative study applied a content analysis approach to key informant interviews, focus groups and field notes to explore the context for implementing the Canadian Association for Psychosocial Oncology (CAPO) fatigue guideline [13]. A previous systematic guideline search and appraisal using the AGREE-II instrument identified the CAPO guideline as suitable for CRF assessment and management worldwide [18]. Our study was conducted during 2018–19 and procedures were approved by the Peter MacCallum Cancer Centre Human Research Ethics Committee (LNR/18/PMCC/205). A project steering committee including the authors, a consumer, a senior oncologist, director of allied health, physiotherapy department head and an implementation researcher provided advice and oversight to the study.

### Participants and recruitment

Multidisciplinary HPs in relevant senior organisational roles with knowledge of current health care delivery, systems and processes at an Australian cancer centre were invited purposively by email to participate in key informant interviews. Focus group participants were invited via emails to discipline heads, presentations at team meetings and advertising in internal communications i.e., convenience sampling. Interested participants were sent an online poll to indicate their availability. Many HPs at the cancer centre were former colleagues of the lead researcher (EP), with knowledge of their CRF research and oncology practice background.

Consumers with experience of CRF were invited to participate in a focus group or interview via information leaflets in outpatient waiting areas, and e-newsletter to a consumer register. Specific culturally and linguistically diverse groups were also approached by a cancer centre consumer liaison officer.

#### Inclusion criteria

##### 1. Health professionals

Registered medical, nursing or allied HPs at Peter MacCallum Cancer Centre (Peter Mac) with skills and experience in delivery of cancer care.

##### 2. Health administrators

Professionals in administration, management or policy roles within Peter Mac.

##### 3. Consumers

Aged 18 + , with any cancer diagnosis, treatment stage or comorbidities, who identified as having experienced CRF. Exclusion criterion: Inability to complete study tasks including consent due to cognitive barriers.

Interview and focus group participants signed informed consent before data was collected. Participants completed demographic details on a study registration form. This included phone or email, year of birth, sex, education, current occupation, cancer diagnosis, treatments, experience with CRF, professional discipline and practice experience, as relevant. The HPs could attend more than one session and were allocated to sessions according to their availability and topic relevance. See Additional Material 1 for optimal group membership.

Additionally, individuals and teams from occupational therapy, clinical psychology, speech pathology, nutrition, day chemotherapy, radiotherapy nursing, haematology and palliative care were consulted informally about their current practice and ways to implement the fatigue guideline. Project team members recorded field notes from these informal meetings or observation. Existing documents related to internal systems and processes, or screening, assessment and interventions for CRF were included as field notes.

In this qualitative study, our pragmatic target sample size was approximately 50 diverse participants including consumers, due to project funding and time constraints. With purposeful sampling and iterative processes, this number was considered sufficient to identify critical issues [19]. An ideal focus group size is six to eight participants [20]. To allow for dropouts, eight to 10 participants from relevant disciplines and services were recruited for each focus group. Formal individual interviews and informal discussions at team meetings provided additional perspectives and increased data richness [21].

### Interview and focus group procedures

Four HP focus groups explored existing practice, barriers and facilitators for different key CRF guideline recommendations: fatigue screening, patient education, fatigue assessment and physical activity. A consumer focus group discussed their experience of, and preferences for CRF management. Interviews and focus groups were led by one female post-doctoral researcher (EP), previously employed as a senior occupational therapist at Peter Mac for over 15 years, with extensive interview experience. This prior relationship facilitated participant trust and engagement with the project. The lead researcher was aware of potential biases due to familiarity with the work setting. She checked assumptions and interpretation routinely with the research team and other stakeholders. A female allied health-qualified research assistant co-facilitated the focus groups and recorded field notes. One research assistant was a doctoral candidate and experienced physiotherapist at Peter Mac and the second, a recently graduated nutritionist. Interviews and focus groups were up to 60 and 75 min in duration respectively and were audio-recorded. A core semi-structured interview schedule was developed, guided by the CFIR domains: characteristics of the intervention (guideline), the inner (organisation) and outer (consumers, society, health policy) settings, individual health professionals’ characteristics and implementation strategies [8]. The schedule was adjusted to match each interviewee’s practice area, and focus group topics. Background information including the CAPO fatigue guideline algorithm and recommendations was provided and used during the sessions. See Additional file 1 for the interview guides, including development information.

### Data analysis

Descriptive statistics were used to summarise participant demographics. To accelerate the progress of the project, a rapid content analysis approach was used for interview and focus group data. Rapid analysis is a method of qualitative investigation that is particularly suited to designing implementation strategies when there is a time constraint [22]. Three concepts characterise rapid analysis: (1) a system perspective, (2) triangulation of data, and (3) iterative data collection and analysis [22]. The rapid analyses used methods described by McNall and Foster-Fishman [23]. To reduce potential researcher bias and increase the study’s validity, two researchers made detailed notes from recordings of each focus group and interview. Concurrently, the notes were coded as direct quotes, paraphrasing or researcher hypothesis. Three researchers cross-checked notes and resolved discrepancies through discussion [23]. Findings were discussed in depth within the team and with some individual stakeholders. To balance the research team’s interpretation, other steering committee members provided practical and theoretical appraisal of results. Summary statements for each question or topic were created i.e., deductive content analysis. These were triangulated with field notes to increase validity and identify barriers, facilitators and potential strategies for CRF guideline implementation. Data saturation was tested via continual feedback and discussion with key participants and teams throughout the project to identify critical factors that would support or prevent guideline use. Reporting adheres to the COREQ checklist [24].

## Results

Five consumers and 31 multidisciplinary HPs participated in eight individual key informant interviews and five focus groups. One HP and one consumer participated via telephone; all others participated in person at the cancer centre. Two students observed one HP interview. Seven HPs and one consumer expressed interest in attending a focus group but were unavailable for a scheduled session. The HP participants were predominantly female (84%), had a median age 43 years (range 24–68) and 10.5 years cancer services experience (range 1.5–32). Most were nurses (55%). Two male and three female consumers participated, having a median age of 59 years (range 43–65) and 5 years (range 2–9) since their diagnosis of four cancer types. Table 1 shows further demographic details.

**Table 1:** Demographic details of focus group and interview participants

Health Professionals *n* = 31			Consumers *n* = 5		
	Median (range)			Median (range)	
Age (years)	43	(24–68)	Age (years)	59	(43–65)
Years since qualification	16	(2–43)	Years since diagnosis	5	(2–9)
Cancer services experience (yrs)	10.5	(1.5–32)	-		
	**n**	**%**		**n**	**%**
**Gender**					
Male	5	16.1	Male	2	40.0
Female	26	83.9	Female	3	60.0
**Area of work**^a^			**Cancer type**		
Acute	14	27.5	Breast	2	40.0
Ambulatory/Outpatients	22	43.1	Melanoma	1	20.0
Day Chemotherapy	9	17.6	Bladder	1	20.0
Community/Home	3	5.9	Abdominal	1	20.0
Satellite campus	3	5.9			
**Discipline**			**Occupation status**		
Nursing	17	54.8	Working	2	40.0
Medicine	4	12.9	Unemployed	1	20.0
Allied HP Total	8	25.8	Retired	2	40.0
Exercise Physiology	1	3.2			
Nutrition	1	3.2			
Occupational Therapy	1	3.2			
Physiotherapy	3	9.7			
Social Work	1	3.2			
Speech Pathology	1	3.2			
Executive	1	3.2			
Radiation Therapy	1	3.2			
**Highest level of education**^a^			**Highest level of education**		
Secondary school	0	0	Secondary school	1	20.0
Undergraduate Degree	3	9.7	Undergraduate Degree	3	60.0
Graduate Certificate/Diploma	12	38.7			
Masters	1	35.5	Masters	1	20.0
Doctorate / PhD	2	6.5			
Specialty Medical Training	3	9.7			
**Self-rated fatigue expertise**			**Experience of CRF**^a^		
Limited	10	32.3	During Treatment	5	100.0
Moderate	17	54.8	After Treatment	4	80.0
Advanced	4	12.9	Currently	2	40.0

^a^More than one response could be selected

Composition of the interviews and focus groups is detailed in Additional file 1. Nurses included chemotherapy, specialist, palliative care and research nurses. Doctors included a pain specialist, one medical and two radiation oncologists. Four HPs participated in more than one session.

### Barriers and enablers to implementing the CAPO fatigue guideline in ambulatory care

**Fig. 1:**
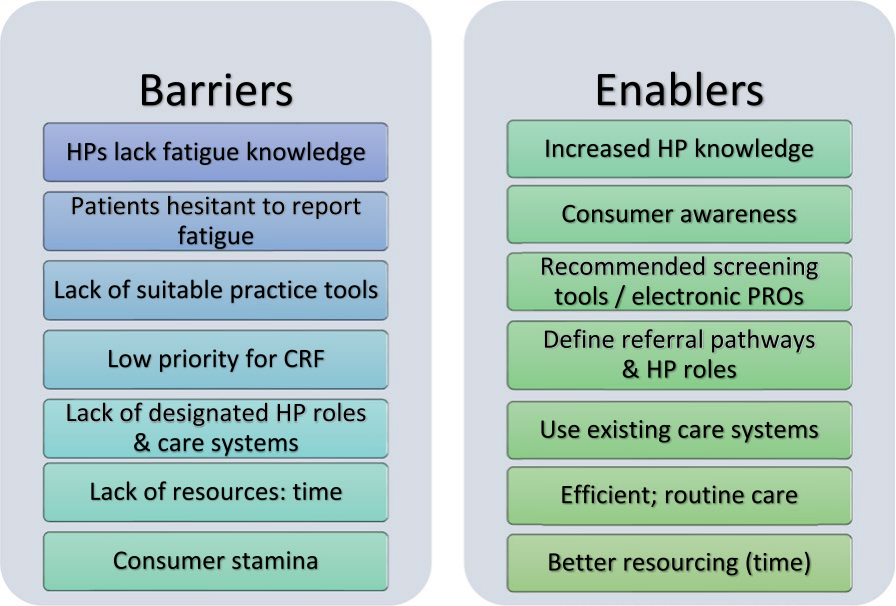
Barriers and enablers to implementing fatigue guidelines

Key barriers to address, and enablers to optimise use of the CAPO fatigue guideline are depicted in Fig. 1. A critical barrier to consistent and comprehensive fatigue management for all HPs was limited time. Most HPs other than occupational therapists felt they lacked adequate fatigue knowledge and resources to support practice. Enablers were alignment of fatigue management with current care processes such as symptom screening, accessible education and simplified practice tools. A further practice barrier was fatigue management not being considered part of the care pathway: *‘It’s not usual care, it’s Unusual care, while it should be usual care’* (Nurse, interview). For consumers, reduced stamina and cognitive abilities, coupled with a perception that HPs did not recognise or prioritise their fatigue were barriers to self-advocacy. Addressing fatigue prevention and management by integrating with treatment, tailoring to individuals’ needs, involving caregivers and using telehealth were consumer-enabling approaches. Table 2 summarises the barriers and enablers to implementing the CAPO fatigue guideline reported by study participants, aligned to four CFIR domains [8]. The fifth CFIR domain—*Implementation strategies –* includes potential actions to reduce barriers and harness enablers to implementation.

**Table 2:** Barriers, enablers and strategies for implementation of CAPO fatigue guideline using CFIR

CFIR domain	Issue / Barrier	Potential solutions / enablers	Implementation strategy (CFIR domain 5)
Inner setting	Systems: Lack of clarity about who should screen for fatigue Systems: Fatigue a low priority and not considered usual care	Build fatigue screening into routine symptom checks	Align fatigue screening with existing screening procedures e.g., chemotherapy, radiotherapy, nutrition screening
	Resources: Lack of time for screening	Encourage patients to self-screen and report symptoms	Simplified patient fact sheet^a^ in chemotherapy education pack and on website
	Systems: Lack of consistency of fatigue screening methods	Develop suite of screening tools to identify mild-moderate-severe fatigue	Resources – screening guide^a^, flip card and lanyard attachment for fatigue
	Systems: No documentation field or prompts for fatigue screening	Medical record form field to record fatigue	Include field in electronic medical record templates
	Workload: Reluctance to screen fatigue due to lack of time to follow up (nurses, doctors)	Develop a stratified assessment with immediate and delayed follow up actions Self-completed patient checklist of contributing factors	Resource – fatigue assessment and referral guide^a^. with second part by telephone or community HP / GP Resource – patient fatigue health checklist
Intervention characteristics	CAPO algorithm is difficult to interpret	Re-design algorithm for mild, moderate, severe levels using ‘traffic light’ system	Resource – one-page fatigue management guide with principles for mild, moderate & severe fatigue^a^
	Lack of practice resources	Develop accessible practice resources	Clinical toolkit including screening, assessment and management guides^a^
	Physical activity guideline is unsuitable for moderate to severe fatigue	Modify physical activity recommendations for moderate & severe fatigue	Resource – Introducing exercise for non-exercise HPs^a^
Individual (HP) characteristics	Lack of knowledge of follow up actions	Clear care pathways using a ‘traffic light' system Training in fatigue screening and management for nursing, medical, allied health and AHAs	Resources – fatigue management and referral guides^a^ Online training in fatigue facts, screening, assessment and management
	Culture: Fatigue management is not considered usual care: *it’s unusual care*	Educate HPs that *something can be done*	Awareness campaign – buttons and modelling / visibility in clinics
Outer setting	Limited patient stamina for longer appointment	Assess fatigue in stages including telehealth follow up	Resources – patient self-assessment for review with HP^a^; assessment guide^a^
	Individual preferences for fatigue rating methods	Different options for rating fatigue, including achievements	Resource – fatigue screening tools^a^
	Consumer reluctance to report fatigue due to further questionnaires and lack of solutions	Educate consumers to self-report and that *something can be done*	Resource: Consumer fact sheet^a^

*AHA* Allied Health Assistant, *CAPO* Canadian Association of Psychosocial Oncology, *GP* General / primary care practitioner, *HPs* Health professionals

^a^For practice resources / clinical toolkit please contact author

### Staff perceptions of fatigue management in practice

Application of the CAPO recommendations for cancer fatigue screening, assessment and management was inconsistent across Peter Mac, with a lack of clarity around whose role it was. Occupational therapists provided fatigue management in their routine practice, and received most referrals from palliative care staff. Fatigue was recognised by HPs as a problem, but during medical encounters there was barely sufficient time to address key disease concerns; while nurses were screening and managing multiple physical and psychosocial issues. Despite recognising fatigue as a key issue, doctors and nurses were often hesitant to screen or ask about fatigue, due to lack of time to follow up or because they were uncertain what to do next e.g., where to refer. This was explained in the fatigue education focus group: ‘*The thing is, if you ask the question and then you get the answer – you’ve gotta do something about it. So sometimes … I won’t ask the question because I don’t know if I can do anything about it’* (Nurse, focus group).

Fatigue management education available for HPs comprised a little known 3-page fatigue management guide ‘Follow up of survivors with cancer-related fatigue’ on the Peter Mac external website, and bespoke online training for occupational therapists. Policy and procedure documents were limited to a precinct document that included management of fatigue in terminal care. Consumers and HPs stated existing patient information was often too long or detailed for people with significant fatigue. Details of how current fatigue management practice aligned with the CAPO fatigue recommendations are shown Additional file 2.

### Consumer experience of fatigue management

Consumers with fatigue had low stamina for travel, waiting and lengthy consultations. When fatigue is a problem, extra questions or tests could be overwhelming due to cognitive changes and exhaustion. ‘*When fatigue was high it caused an inability to multitask and follow long discussions – caused me a lot of distress. What is wrong with me? … A lot of my cognitive skills just shut down’* (consumer #5, female age 43).

Screening and acknowledgement of fatigue was welcomed by consumers, but it was unusual: ‘*Fatigue isn’t usually mentioned, it’s like it’s a given and that’s that.’* (consumer #1, female age 59); ‘*this is not imaginary, this is debilitating, and it needs attention.’* (consumer #3, male age 65). However, screening could also be distressing, with some people under-rating their fatigue. One participant reported under-rating her fatigue due to feeling inadequate in self-management. ‘*Whenever I’ve got a zero to ten, I tend to under rate. I don’t ever want to be a 10. Cause that means to me that I’m sort of not coping and I don’t want to admit that.’* (consumer #1, female age 59). Another consumer lowered her fatigue severity rating due to perceptions of staff time pressure: *‘I was asked to fill out a scale and … I circled a 6 [out of 10]. And [the HP] said ‘if it’s more than a 4 then you have to do the other side’ – and there was a few things on the other side. So I changed it to a 4…. She kind of was indicating she didn’t want to take any more time, so change it to a 4…’* (consumer #2, male age 60).

## Discussion

Implementing guidelines for fatigue screening, assessment and management needs careful planning with consideration of consumer, practitioner and system/organisational perspectives [15]. Our investigation identified a range of barriers and enablers to implementing the CAPO fatigue guideline at an Australian comprehensive cancer centre. Predominant HP barriers to fatigue management related to lack of knowledge, time and practice resources, such as standard screening methods. These caused HPs to avoid asking about fatigue. Surprisingly, 87% of HPs rated their CRF expertise as ‘limited’ or ‘moderate’—despite having a median of 10.5 years’ oncology experience. This suggests access to fatigue training or opportunity to use the knowledge are lacking. When consumers did experience fatigue screening, lack of follow up discouraged them from raising the topic again, or to downgrade their fatigue severity rating. These findings are not unique to Australia, with knowledge and system barriers coupled with poor fatigue communication previously reported [9, 16, 17, 25–28]. A recent Canadian study found major themes which accounted for the CRF knowledge-practice gap were *“a perfect storm”* and *“a breakdown in communication”* [17], both themes strikingly congruent with our findings. “*A perfect storm”* characterised inadequate HP knowledge of CRF guidelines in a setting of system barriers and limited funding; while “a *breakdown in communication”* involved HPs avoiding or normalising fatigue, leaving patients feeling helpless and dismissed [17].

The interrelationship of the barriers identified highlights the complexity of implementation. For the HP, it begins with awareness and knowledge of the guideline [29]. Clearly HP education is required, but acquisition of knowledge and expertise takes time, a limited commodity in today’s health care context. Adding new tasks for managing fatigue on top of education in an already time-poor context increases pressure on HPs. Further, guidelines are often sparse in detail and notoriously lacking in practice resources to assist delivery to the particular patient [30], leaving interpretation and decisions to the HP – who may lack adequate knowledge [31]. The current and earlier studies [9, 18] reveal the CAPO fatigue algorithm has limited clinical utility in its current format and practice resources such as screening tools, assessment and management guides are needed. Lack of both time and knowledge about CRF then results in HPs avoiding the issue and leaving patients to manage on their own [17]. Multiple strategies are needed to overcome these barriers.

Along with these barriers, we identified opportunities to implement CRF guidelines. It is noteworthy that both HPs and consumers in our study described time constraints in the clinic as barriers to fatigue management. Therefore, time-efficient strategies for fatigue screening and assessment should be prioritised. Documentation prompts such as an electronic medical record field for fatigue could contain a link to screening, assessment and management resources. Occupational therapists and other allied HPs with specialist knowledge and holistic assessment practices could lead comprehensive fatigue management. Because such cancer specialist HPs are limited in number, accessible education enabling local HPs to provide cancer fatigue management is essential for equitable cancer care.

Translation of CRF guidelines into practice has typically been slow globally [15]. This may be in part because prevention and management of fatigue is not commonly identified as a priority at organisational and policy levels, remaining forever in competition with other initiatives for cancer treatment [15]. Government supportive care policies without adequate funding or resources to provide care cannot be implemented equitably. We endorse Berger and colleagues’ proposition that innovative care models such as telehealth and harnessing e-health records are needed to adequately assess and manage cancer fatigue and other symptoms [15]. It is time to shift research focus to effectiveness-implementation hybrid trials [32] exploring novel ways to implement cancer fatigue guidelines that are sustainable for both provider *and* consumer. Approaches already being evaluated include stepped-care approaches [33–35], telehealth or online home based programs [36–38] and comprehensive assessment clinics [39].

This study had several strengths and limitations. Including a broad range of end-users in preliminary scoping work enabled a 360-degree approach to barriers and enablers to guideline implementation, and ‘ownership’ in the process of practice change [40]. However, some groups such as senior managers and doctors were under-represented and may have offered different views. Additional consumer input may have strengthened findings however our consumer findings were congruent with previous studies [9, 17]. Stakeholders’ insights into barriers and enablers in four CFIR domains point to strategies to enhance the success of guideline implementation efforts [41]. Rapid analysis of qualitative data is an established practice in crisis situations, that provided information in real-time [23] to accelerate the next phase of guideline implementation. Traditional or inductive qualitative analysis may have produced different results.

Only some of our findings such as documentation are site-specific. Lack of time is a global issue that needs to be addressed at national policy level. However, lack of practice resources and knowledge previously reported in local and international studies [9, 16, 17, 42] remain barriers to best practice fatigue management and these could be developed to be broadly applicable. The current study used the CFIR to enrich knowledge by identifying strategies to overcome implementation barriers related to the intervention (CRF guideline), individual, inner and outer contexts [8]. For successful implementation, a holistic approach is needed to tackle barriers in all CFIR domains within a local political context.

## Conclusions

Our results underscore a critical need for practice tools and health professional education to support CRF guideline implementation. Cancer fatigue training and/or management should be accessible and time efficient for both HP and consumer and guideline processes should be integrated with existing processes. Strategies for guideline implementation focusing on sustainability should be trialled. To achieve adequate and equitable management of CRF, the scope of funding for cancer care must reflect best practice supportive care guidelines.

## Supplementary Information

**Additional file 1.**

**Additional file 2.**

## Data Availability

De-identified focus group summaries are available upon reasonable request to Dr Pearson.

## Abbreviations

CAPO: Canadian Association for Psychosocial Oncology
CBT: Cognitive behaviour therapy
CFIR: Consolidated Framework for Implementation Research
CRF: Cancer related fatigue
HP: Health professional
NRS: Numeric rating scale
