# Supplementary material for: Identifying strategies for implementing a clinical guideline for cancer-related fatigue: a qualitative study
Source: BMC Health Serv Res. 2023 Apr 24;23:395. doi: 10.1186/s12913-023-09377-9 (PMC10127293; doi:10.1186/s12913-023-09377-9)
Supplement: Supplementary file 1 — Additional file 1. [file 12913_2023_9377_MOESM1_ESM.docx]

**Overview of focus group topics**

The purpose of the focus groups will be to establish acceptable procedures and strategies for delivering CRF management at xxx [1]. Health professionals who will implement the guideline recommendations will contribute to propose, evaluate and determine strategies and tools for delivery of evidence-based CRF management at xxx In parallel, one or more consumer focus groups will be held to ensure consumer information and service delivery needs are met [2].

As the topic of the focus groups differ, the questions are generic with <Topic X> indicating a main topic / guideline recommendation as listed below. After interviews or discussion with relevant professionals, the CAPO recommendations *Treatment of contributing factors* and *Cognitive behaviour therapy* were not prioritised for focus groups.

Table 1: Target participants for focus groups and interviews

| **Main <topic> / CAPO recommendation:** | **Target Participants (focus group &/or interview)** | | |
| --- | --- | --- | --- |
| 1. **Screening for presence of fatigue** | Admissions clerk  Clinical nurse coordinator (CNC)  Ward nurse | RT nurse  Nurse manager  Social worker  Medical Registrar | Occupational therapist  Medical records personnel |
| 1. **Focused and comprehensive assessment of fatigue** | Medical Registrar  HMO  Consultant  CNC  Dietitian | Palliative care  Late effects OP nurse  Pharmacist  Clinical Psychology | Domiciliary nurse  OT/PT  Hospital information  Telehealth administrator |
| 1. **Fatigue education including complementary / herbal medicines** | Nurse  CNC  OT  Survivorship Centre &/or wellness centre manager | PT  Palliative care nurse  Pharmacist  HMO / Registrar | Social worker  Pastoral / meditation  Education officer  Telehealth |
| 1. **Physical activity** | PT  Exercise Physiologist | OT  Nursing | Survivorship  Ontrak (AYA) |
| 1. **Consumer needs and preferences re screening, assessment and fatigue education** | Consumers – focus group and interview | NESB consumers |  |
| AYA - Adolescent and young adult service; CNS – Clinical nurse specialist; Diet – Dietitian; EP – Exercise Physiologist; HMO – Junior doctor; MSW – Medical Social Worker; NESB – non-English speaking background; OP – outpatient; OT – Occupational Therapist; PT – Physiotherapist; RT – Radiation therapy; SP – Speech Pathologist | | | |

Table 2: Actual interview and focus group participants

| Type of interview | Allied Health | Nurses | Doctors | Other | Total |
| --- | --- | --- | --- | --- | --- |
| Key informant interviews | 1 MSW  1 OT | 4 | 1 | 1 Consumer | 8 |
| **Focus group topic** |  |  |  |  |  |
| Fatigue experiences and preferences | - | - | - | 4 Consumers | 4 |
| Fatigue screening | 1 Diet | 4 | 2 | 1 Radiotherapist | 8 |
| Fatigue assessment | 1 SP | 7 | 1 |  | 9 |
| Fatigue education | - | 6 | - | 1 Executive | 7 |
| Physical Activity | 1 EP, 1 OT, 3 PTs |  |  |  | 5 |
| Diet – Dietitian; EP – Exercise Physiologist; MSW – Medical Social Worker; OT – Occupational Therapist; PT – Physiotherapist; SP – Speech Pathologist | | | | | |

**Materials needed:**

- PICF, consent forms x10, registration form x 10
- 12 Laminated copies [of CAPO fatigue algorithm](https://www.capo.ca/resources/Documents/Guidelines/6.%20Algorithms%20for%20Cancer%20related%20Fatigue.pdf)
- Whiteboard or butcher’s paper and appropriate pens
- PowerPoint presentation – overview of CAPO fatigue, Topic X recommendations & data
- Post-It notes, paper and pens
- Audio-recorder

**Focus group general structure (60 mins)**

1. *Introduce facilitators and answer questions about the PIC and consent signing (10 min)*
2. *Introduce purpose / topic of focus group (10 mins)*
3. *Review of project findings to date (20 mins)*
4. *Developing strategies to increase guideline feasibility (25 mins)*
5. *Gaining consensus and prioritising strategies (15 mins)*
6. *If sufficient time - final brief exercise: THE LAST WORD [3] and what next*

**Health professionals – key informant interviews**

Key Informants were identified due to their professional knowledge and role related to specific recommendations in the CAPO fatigue guideline, or their status as “opinion leaders”. The purpose of key informant interviews is to obtain information about the acceptability and feasibility of an intervention within an organisational setting (Ayala & Elder, 2011).

Questions and text is a guide only. Not all questions were asked in every interview. For example, one interview on Psycho-education may focus on fatigue self-management and education and another would consider CBT and mindfulness.

As the focus of the interviews will differ depending on professional scope, the questions are generic with <Topic X> indicating a main guideline category.

Interview participants – Clinical nurse specialist, palliative care nurse specialist, day chemotherapy nurse x2, senior occupational therapist, medical consultant (pain), and social worker

| **Main Topics / <Topic X>** | **Key Informants’ main topics** |
| --- | --- |
| 1. Screening for presence of fatigue and referral | Clinical Nurse Coordinator (CNC)  Day chemotherapy nurse  Social worker |
| 1. Assessment of fatigue and contributing factors | CNC  Day chemotherapy nurse  Medical consultant (pain)  Senior Occupational therapist |
| 1. Treatment of contributing factors |  |
| 1. Psycho-educational interventions | CNC  Senior Occupational Therapist  Day chemotherapy nurse  Social worker |
| 1. Exercise interventions | Senior Occupational Therapist |

**~~~~~~~~~~**

**Interview guide – key informants**

1. **Introduce self and answer questions about the PIC and ensure consent is signed.**

“Hi We are <names> from Allied Health and Cancer Experiences Research. Our roles in this project are Principal Investigator / Research Assistant for this project funded by the Victorian Cancer Agency. This project aims to provide better management of cancer-related fatigue, to improve outcomes for cancer survivors.”

“Thanks for agreeing to be interviewed for this project. Have you had an opportunity to read the Participant Information Sheet? Before you sign the consent form, do you have questions about being involved in this study?” (Confirm the participant agrees to audio recording)

After consent is signed, start recording.

1. **Introduce purpose / topic of interview**

“The purpose of today’s interview is to find out about what is currently happening, and what barriers and potential facilitators exist to implementing fatigue guideline recommendations at xxx. Before we get into that can you please briefly describe your professional background and your clinical &/or managerial role here.”

Prompts: “How long have you worked here / in oncology / been in that role?”

“What is your level of autonomy in your day to day work?”

1. **Outline guideline and introduce <topic X>**

“The Canadian Association for Psychosocial Oncology guideline for cancer-related fatigue (CRF) is a complex intervention and contains recommendations for fatigue screening, assessment and management. From the algorithm *(indicate document on table)* you can see there are a number of recommendations. Today we will focus on <topic X> as a component of the whole guideline.”

1. **Overall view of fatigue management at xxx.**

“What do patients and external colleagues expect of fatigue management at xxx?”

“Do you think current management of CRF at this Centre meets the needs of most patients?”

“How important is cancer fatigue management in relation to other symptoms at xxx?”

“Do you think there is a need / appetite for a change of practice for managing CRF?”

“How do you feel about implementing fatigue guidelines?”

1. **Current practice for <Topic X>**

*Explain the CAPO recommendations for Topic X: Use printed material as prompt and read out recommendation/s.*

E.g. Topic <CAPO Recomendations for Psychoeducation>

- All patients are likely to benefit from routine fatigue education with emphasis on self-care, coping techniques, energy and activity management.
- Education should be targeted to fatigue with a minimum of 3 sessions
- Cancer services should promote access to multi-component, group psycho-education programs targeted to self-management of fatigue
- Referral to experts trained in CBT specifically targeted to fatigue should be offered to survivors with chronic cancer fatigue
- Preliminary evidence that mindfulness based interventions may improve fatigue
- Reading, games, music, gardening, experience in nature may distract from fatigue

“Are there any policies or procedures related to <Topic X>?”

“How is <Topic X> currently done at PMC and by whom?”

“What forms, tools and systems are used to do <Topic X>?”

“How is <Topic X> documented?”

1. **Barriers to implementing guideline and <Topic X>**

“What do you see as barriers to implementing the fatigue guidelines at Peter Mac?”

Prompts:

“In the past when new processes have been introduced, what sorts of issues have arisen?”

“How do you think health professionals would accept an evidence-based fatigue guideline?”

“What patient factors could act as barriers?”

“In relation to <Topic X>, what barriers can you foresee (in your area)?”

Prompts:

“What practical issues can you foresee with <Topic X>?”

“How feasible are the recommendations?”

“How well does <Topic X> align with symptom management guides and priorities?”

“Can you comment on the complexity and feasibility of <Topic X>?”

1. **Enablers for implementing CAPO guideline**

“Success in implementing the CAPO fatigue guideline will depend on a number of elements such as attitudes towards the intervention, organisational culture, simplicity, fit with current practice and consumer feedback”.

“What existing factors of systems will facilitate implementation of <Topic X>?”

“What strategies or conditions will help to implement <Topic X>?”

Prompts:

What additional details would you need to follow these recommendations?

How well does <Topic X> align with current care delivery?

How could <Topic X> be streamlined for efficiency?

What forms, materials and systems are needed for <Topic X>?

How could documentation about <Topic X> be facilitated?

“What HP education/training is needed for <Topic X>?”

“Which health professionals should complete <Topic X> and what credentialing is needed?”

“How could other systems of care (e.g. self-management, tele-health, online) be used for <Topic X>?”

1. **Conclusion of interview**

“Do you have any further comments about implementing the CAPO guidelines or <Topic X> at xxx?”

“Can you suggest any individuals in <your area> to invite to a focus group on <Topic X, Y or Z>?”

“Thank you for your time today. If anything else comes to mind after this interview, please do let me know either by email or phone / message.”

**Health professionals –** **Focus Group guide**

As the topic of the focus groups will differ, the questions guide is generic with <Topic X> indicating a main topic / guideline recommendation as listed below.

**Focus group 1 – Physical activity for fatigue management (60 min)**

Participants – Occupational therapist, exercise physiologist, physiotherapists x3

**Focus group 2 - Screening for the presence of fatigue (60 min)**

Participants – Dietitian, radiation oncologist x2, radiation therapist, nurse x4

**Focus group 3 – Assessment of the fatigued patient (60 min)**

Participants – Social worker, medical oncologist, nurse x6, speech pathologist

**Focus group 4 – Patient fatigue education (60 min)**

Participants – Nurse x6, executive

**~~~~~~~~~**

1. ***Introduce facilitators, answer questions about the PIC and ensure consent is signed. (10 mins)***

“Hi I am <name> and this is <name> from the Allied Health and Cancer Experiences Research Departments. My role in this project is Principal Investigator / Research Assistant for this project funded by the Victorian Cancer Agency. This project aims to provide better management of cancer-related fatigue (CRF), to improve outcomes for cancer survivors. Previous studies have indicated that about 20% of oncology health professionals in Australia followed a guideline for CRF [4]. Consumers reported fatigue as an unmet need [5, 6], and both health professionals and consumers perceive a lack of expertise in managing CRF [4, 7]. Use of evidence-based guidelines for CRF management has the potential to improve outcomes for cancer survivors, but we don’t know how feasible or effective they are. In partnership with staff and consumers, this project will determine how to implement evidence-based fatigue management at xxx.”

“Thanks for agreeing to participate in a focus group to shape this project. We are looking for solutions to increase the feasibility and acceptability of the CAPO fatigue guidelines.

Before commencing today I need to ensure that you all have read and understood the Participant Information Sheet. Are there any questions about being involved in this study?” (Confirm the participant agrees to audio recording).

I want to remind you that the information discussed today will be recorded and used as research data. Your supervisor will not be informed about what you say individually however group results will be reported. I hope you discuss this more widely with other colleagues, but please respect other participants by ensuring names or disciplines are not used.”

After consent is signed by participant and researcher, begin recording.

1. ***Introduce purpose / topic of focus group (10 mins)***

*Preamble.* “You and your colleagues will be the users of this guideline, and it is important that strategies to simplify, streamline and enhance the guideline are acceptable to users. Importantly, consumers will provide separate input about how their fatigue management care should be delivered.

*“*Before this focus group we interviewed a number of Key Informants about current policy and practices related to the fatigue guideline.

“The main purpose of today’s focus group is to discuss ways of minimising barriers to implementing fatigue guideline recommendations at xxx. We will be focusing on <Topic X> within the context of the guideline algorithm *(indicate printed material that is in front of participants – algorithm).*

*Show brief PowerPoint overview of the CAPO guideline algorithm and full <Topic X> recommendations.*

1. ***Review of project findings to date (20 mins)***

“First we will review findings to date about current policy, practice, and identified barriers to implementing <Topic X>. These include findings from recent research and interviews”

*PowerPoint slide: dot points on current practice and barriers. [This will be developed following Key Informant Interviews] [4, 7]*

- **“How do these findings fit with your experiences? Do you have anything to add?”**

Prompts:

Can you add detail about workplace culture / attitudes?

Do you think your colleagues would welcome this guideline to help their practice?

Do you perceive a need for better coordination of CRF management?

- **“Which barriers do you think are key to success of implementing <Topic X>?”**

*Write barriers on whiteboard or butcher’s paper sheet*

- **“What would you say are the top three?”** *Asterisk barriers and check most agree. (Note areas of disagreement)*

1. ***Developing strategies to increase guideline feasibility (25 mins)***

“Ok let’s agree on the top three barriers you have identified. *These will also be written on whiteboard.*

- **How could Barrier 1 be minimised?**

Prompts as appropriate to barrier:

“Can you suggest ways of integrating fatigue into current symptom management practice?

“What tools could make <Topic X> easier?
E.g. proforma, checklist, educational handouts, flow chart, instructions etc”

“How can we increase awareness and HP knowledge about fatigue management?

“How could we clarify who is responsible for <Topic X>?

“How can we engage local ‘champions’ in fatigue guideline use?”

“What additional resources could enable <Topic X>?”

“Do you feel you need extra training to implement <Topic X>?”

“How might <Topic X> be stratified for different levels of fatigue?”

“What changes are needed to implement <Topic X>?”

- **How could Barrier 2/3 be minimised?**

1. ***Gaining consensus and prioritising strategies (15 mins)***

*Activity:* “To ensure everyone contributes equally to prioritising strategies for implementation of <Topic X>, we are going to use a modified consensus method called the “Nominal Group Technique”[8].

“Everyone has 10 votes for the various strategies. You must use all your 10 votes but you can give them in any way you like. For example, 4+3+2+1=10 or 5+1+1+1+1+1=10 or even all 10+0 if you feel strongly about one strategy. Write each strategy you are voting for on a separate post-it note then how many votes you give it. Check they add up to 10 then pass them in together.”

*Hand out Post It notes. Allow a few minutes for voting. While people are voting, photograph whiteboard and tidy up so that votes can be written in. When all the votes are in, write them up on the board and totals. Keep notes from one person bundled together to check later.*

- **How do these priorities sit with you? Is there anything that anyone strongly disagrees with?**

1. ***If sufficient time - final brief exercise: THE LAST WORD [3]***

Activity: “To ensure everyone has a say about future actions, we would like you to write down your ‘take-home messages’ about what should be done. These will be analysed alongside the recording.”

- **“What could you do to influence your colleagues to follow the CAPO fatigue guideline?” &/OR**
- **“If you could tell the Peter Mac policy-makers one thing to do to improve CRF management, what would it be?”**

“We are out of time. I will send around the proposed actions by email to give you a chance to comment after the focus group, in case you think of important things that we have not considered”. Please reply simply “OK” if you agree, or add a comment if you have additional thoughts, or disagree.”

“As the session might not be transcribed in full immediately, it may be some time before the full transcript is available. In the email we will ask you whether you would like a copy when it becomes available to review.”

“Thank you so much for your time. I can be contacted by email or phone if you would like to discuss this further.”

**Consumers – focus group / interview guide**

1. *Introduce facilitators and answer questions about the PIC and sign consent. (10 mins)*
2. *Introduce purpose / topic of focus group / interview (10 mins) - presentation*
3. *Impressions of the CAPO guideline (15 min) - discussion*
4. *Developing strategies to increase guideline feasibility (25 mins) - discussion*
5. *Take-home message: The Last Word [3] (10 mins)- written exercise and what next*

**~~~~~~**

1. ***Introduce facilitators and answer questions about the PIC and ensure consent is signed. (10 mins)***

“Hi I am <name> and this is <name> from Allied Health and Cancer Experiences Research. My role in this project is Principal Investigator / Research Assistant for this project. The aim is to provide better management of cancer-related fatigue, to improve outcomes for cancer survivors.”

“Thanks for agreeing to participate in this focus group / interview. We are looking for solutions to increase the feasibility and acceptability of the CAPO fatigue guidelines.

Before commencing today I need to check that you all have read and understood the Participant Information Sheet. Are there any questions about being involved in this study?” (Confirm the participant agrees to audio recording).

The focus group / interview today will last for up to 1½ / 1 hours. We will talk briefly about the Canadian cancer fatigue guideline and some recent research with consumers about using it. Then you will discuss those findings in the context of your experience. Then you will discuss how we could use the guideline in a way that is most feasible for consumers to use. The session will finish with a paper and pen exercise where you write your ‘take home message’.

I want to remind you that the information discussed today will be recorded and used as research data. Your treating team will not be informed about what you say individually however group results will be reported. I hope you discuss this more widely with health professionals and other patients, but please respect other participants by ensuring names or disciplines are not used.”

After consent is signed by participant and researcher, begin recording.

1. ***Introduce purpose / topic of focus group / interview (10 mins)***

*Preamble.* “When introducing changes in health practice, it is important that strategies to simplify, streamline and enhance the guideline are acceptable to users. It is especially important that consumers provide input about how their fatigue management care should be delivered.

*Context. “*We have been consulting with staff about current policy and practices related to the fatigue guideline. They have been advising on how to put the guideline into practice.

“The main purpose of today’s focus group / interview is to discuss ways of minimising consumer barriers to implementing fatigue guideline recommendations at xxx. First let’s look at the main guideline recommendations. *Show brief PowerPoint overview of the CAPO guideline and hard copy algorithm.*

1. ***Impressions of the CAPO guideline (15 min)***

“Unfortunately, we don’t have enough time to discuss your individual experiences with fatigue management. What we will do is talk about the how guideline recommendations can be put into practice in a way that is appropriate and accessible to consumers.

“Some of my research has highlighted issues for consumers using CRF guidelines [4]. These include: [PowerPoint slide]

*Consumers value health professionals who really listen and recognise their fatigue*

*Consumers want to know who to talk to about their fatigue*

*Fatigue management should occur as part of routine care (not an add-on)*

*Consumers want to choose how, when and where they fill questionnaires about fatigue*

*Consumers want information in different levels of detail and in different formats*

*Having fatigue makes everything a lot harder.*

- **Do these findings match with your views? Do you have anything to add?**
- **“Which consumer issues are most important to address when putting the CAPO guideline into practice?”**

Prompts:

1. ***Developing strategies to increase guideline feasibility (25 mins)***

“Several suggestions to improve fatigue management have been made. These include a way of tailoring the process to a person’s fatigue [PowerPoint slide]:

Simple education about fatigue and self-monitoring for all – e.g brief explanation / handout

Self-management for people with mild to moderate fatigue with group or online education

Health professional consultations for people with moderate-to severe fatigue

- **What essential consumer information is needed for everyone with or without CRF?**
  - **How could consumers get information that is at the right time and level for them?**
- **How feasible do you think fatigue Self-monitoring would be for most consumers?**
- **How could consumers with moderate to severe fatigue receive fatigue management?**
  - **How acceptable is Telehealth for someone with moderate to severe fatigue?**

Prompts as appropriate:

“What tools could make that easier?
E.g. patient completed questionnaire, educational handouts, etc”

1. ***Take-home message: The Last Word [3] (10 mins)***

*Activity:* “To ensure everyone has an equal say about future actions, we would like you to write down your ‘take-home messages’ about what should be done. These will be analysed alongside the recording.”

- **What are the most important things to you about how xxx should approach CRF screening, assessment and management?** *[Put up on PowerPoint Slide]*

*At 70-72 minute time point:* Conclusion to session

“Our time is up. We will send you a summary of the focus group / interview and proposed actions. In case you think of important things that we have not discussed, or disagree strongly, please let us know in your reply.”

“I think is important to talk about this outside the focus group / interview. I’d like to remind you not to use participant’s names when talking about a person’s opinions. We will do the same in our reports”.

“Thank you all so much for your valuable time today. I do hope you have found the experience worthwhile. You are welcome contact [researcher] by email or phone to discuss this further.”

**Development of interview guide: Evidence from CFIR domains relevant to guideline recommendations**

| **TOPIC / guideline recommendation** | **Guideline characteristics** | **Organisational context: current practice & policy** | **Health Professional Characteristics** | **External context inc consumers** |
| --- | --- | --- | --- | --- |
| General  (evidence-based) | User-friendly format  Structured  Electronic  Index and appendices – operational detail  Stratification  Brief (e.g. exec summary)  Implementation resources | Org/Management endorsement of guideline: priority  Discipline roles  Integration  Continuity of care  Efficiency  Services available e.g. CBT  Cost/resource measurement  Outcomes - measurement | Credentialing  Education / knowledge / skill  Existing online resources  Attitudes / feasibility  Leadership - Promote guideline use  Take fatigue seriously  Ask the question  Champions | Endorsement – leading cancer organisation  Funding stream / policy  Link to local contacts  Convenience / accessible  Local services  Stratified education / info  Individual / Consumer preference |
| Screening for presence of fatigue and measuring fatigue / impact | In relation to <fatigue screening>, does the guideline clearly state what to do, when to do it and for whom?  What tools would support <fatigue screening>?  Distress thermometer [1]  Validated screening tool.  What should the **cut points** be? (eg 4/7 for mod & severe)  Is there a need to distinguish CRF from other fatigue (eg DICRF)? *Research question.*  Tailoring options  Screening handout | When is <fatigue screening> currently done?  How is it done (tools & doc)?  What is done with screening results (**referral pathways**)?  Who should <screen for fatigue> at Peter Mac?  What changes are needed to implement <fatigue screening>? | What education do health professionals need for <fatigue screening / measurement>?  How should HP <fatigue screening> education be provided?  Who will coordinate <fatigue management> and provide support? | How can consumers **self-monitor** their fatigue/energy level?  What essential **consumer information** is needed about <screening>?  How should this info be provided? |
| Focused and comprehensive assessment of fatigue *(≥4 on NRS*) | What might a **stratified assessment guide** look like?  How should we **measure changes** in fatigue? (Is the **Brief Fatigue Inventory** feasible/sufficient or do we need an objective measure?)  What **tools** are needed for focused and comprehensive assessment of fatigue e.g. guide to discussing fatigue, multi-symptom / assessment and documentation tools?  **Details of assessments** (eg physical examination) and significance of findings.  What **decision making tools** would be helpful (e.g. detail)?  Fitness testing / activity level  Tailoring options | How is **fatigue assessment** currently done at PMC?  Who could conduct **focused fatigue assessment**?  Could a patient questionnaire be used to flag **contributing causes**?  Who should complete **comprehensive fatigue assessment** (including tests)?  How could **fatigue assessment** be done using telemedicine?  How could **fatigue assessment** be streamlined for efficiency? (Stratified)  What **forms and systems** are needed for fatigue assessments?  What referral mechanisms are needed for specialist consults? | What HP education/training is needed to undertake **focused fatigue assessment**?  What HP education is needed to undertake **comprehensive fatigue assessment?**  What credentialing is needed to conduct **focused fatigue assessment**?  What credentialing is needed to conduct **comprehensive fatigue assessment**? | Could consumers complete a questionnaire / checklist for possible **contributing factors**?  How could **fatigue assessment** be done at home / using telemedicine?  Minimise burden of assessment (fewer additional appointments / longer appointments) |
| Treat contributing factors | Information / detail needed?  Pathways?  Tailoring options | Who should coordinate?  Automatic appointment with referral  Integrate with symptom management systems | Rapport, reassurance, caring | Consumer preferences / capacity (fatigue limitations) |
| Fatigue education  *Education: energy management* | Screening handout  Documentation of education  Accessible information to give to patients  Content of education for fatigue prevention, moderate and severe fatigue  Targeted to fatigue - emphasizes self-care, coping techniques, energy, and activity management  Minimum 3 sessions  Tailoring options | Individual education – by whom (role)  Group psycho-education targeted to self-management - about fatigue and general strategies | Education about fatigue, screening and guideline  Education for specific interventions or assessments | Basic, standard, detailed  Relevant to fatigue level  Multiple modalities  Online [2] |
| Promote physical activity | No consensus on Optimal exercise dose  Moderate x 30min x 5+ days  Vigorous x 20min x 3+ days  All PA at lower intensity  Yoga  Referrals high risk & tailored regimes (symptoms)  Advanced ca - supervised  Tailoring options: Guidelines for PA for mild, moderate and severe fatigue levels | Referral systems – internal / external eg initial evaluation then referral close to home  Telehealth approaches | Credentialing: What knowledge and experience is needed to safely promote physical activity?  What is required to prescribe physical activity / exercise?  What considerations are needed for severely / moderately fatigued? | Online / remote options  Community resources  Consumer preferences |
| Pharmacological management - OMIT | CAPO: Insufficient evidence to recommend pharma agents at any stage | Current practice | Education component | Include in education |
| Cognitive behaviour therapy - OMIT | Targeted to fatigue (sleep)  Tailoring options | Referrals to clinicians trained in CBT specifically targeted to fatigue offered to pts and survivors with fatigue  What is currently happening? | Credentialing: What knowledge and experience is needed to offer CBT for fatigue?  Who? | Available services  Online [3] / remote options |
| Complementary therapies - OMIT | Mindfulness-based Tx may improve fatigue  Insufficient evidence:  Acupuncture[4]  Herbal medicine  *Limited evidence for massage, healing touch, relaxation training & hypnosis  Tailoring options | What mindfulness therapies are currently offered? | What are HPs saying to patients about CAM for fatigue? | Consumer preferences |

1. Abrahams HJG, Gielissen MFM, de Lugt M, Kleijer EFW, de Roos WK, Balk E, Verhagen CAHHVM, Knoop H: **The Distress Thermometer for screening for severe fatigue in newly diagnosed breast and colorectal cancer patients**. *Psychooncology* 2016:n/a-n/a.

2. Yuen HK, Mitcham M, Morgan L: **Managing post-therapy fatigue for cancer survivors using energy conservation training**. *J Allied Health* 2006, **35**(2):121E-139E.

3. Abrahams HJG, Gielissen MFM, Goedendorp MM, Berends T, Peters MEWJ, Poort H, Verhagen CAHHVM, Knoop H: **A randomized controlled trial of web-based cognitive behavioral therapy for severely fatigued breast cancer survivors (CHANGE-study): study protocol**. *BMC Cancer* 2015, **15**(1):765.

4. Zhang Y, Lin L, Li H, Hu Y, Tian L: **Effects of acupuncture on cancer-related fatigue: a meta-analysis**. *Support Care Cancer* 2018, **26**(2):415-425.

**A pan Canadian practice guideline for screening, assessment, and management of cancer-related fatigue in adults Version 2-2015**

For full guideline see <https://www.capo.ca/wp-content/uploads/2010/10/CRF_Final.-20150521.pdf>

**Definition**

**Cancer-related fatigue** (CRF) is a “distressing, persistent, subjective sense of tiredness or exhaustion related to cancer or cancer treatment that is not proportional to recent activity and interferes with usual functioning” (1)

| Guideline developer: Canadian Partnership Against Cancer, Canada (2) | | Space for notes / impressions |
| --- | --- | --- |
| Version  Last update  Next update due | Version 2 2015  April 2015  2020 |  |
| Literature search | November 2014 |  |
| Development methodology | Systematic review and ADAPTE (3) |  |
| **Population**: | Adult |  |
| Treatment phase | During and after treatment |  |
| Disease stage | All including end of life |  |
| With CRF | Yes |  |
| Categories of evidence used | **GRADE ratings** (4)  Strong or Weak with High, moderate or low quality evidence.  Also ‘Expert panel consensus informed by guideline evidence’ for some recommendations |  |
| 1. **Screening for fatigue – recommendations - all NCCN level 2A** | |  |
| Population | Adults **with CRF**, any stage of illness |  |
| Screening instruments | Use a valid quantitative measure with established cut-offs (e.g. 0-10 NRS; ESAS; FACT-F; Piper Fatigue Scale) or semi-quantitative tool (Fatigue Pictogram) |  |
| Routine screening for fatigue at regular intervals | Screen for presence and severity at entry to system, specific intervals during treatment, follow up and advanced disease. |  |
| Ad hoc screening | As clinically indicated – changes in disease status or treatment.  Early detection and thorough evaluation recommended specially in elderly. |  |
| 1. **Assessment recommendations - all NCCN level 2A** | | **Notes** |
| Population | Adults with CRF, any stage of illness |  |
| Conduct further assessment of fatigue | For fatigue rated >3 on NRS - focused assessment |  |
| **Focused fatigue assessment** | Quantitative Fatigue measurement tools  Onset, pattern, duration  Associated or alleviating factors  Interference with function  Evaluate disease status:  Risk of recurrence  Review of systems  Type / length of treatment  Beliefs, values and knowledge of fatigue |  |
| **Assessment of treatable contributing factors** | Pain  Anxiety  Depression  Sleep disturbance  Nutritional deficits  Deconditioning /wasting  Anaemia  Decreased physical activity / function  Infection, fever  Nausea |  |

| **Assessment recommendations - all NCCN level 2A** | |  |
| --- | --- | --- |
| **Assess comorbidities** | Cardiac dysfunction / cardiomyopathy  Endocrine dysfunction (hypothyroidism, hypogonadism, adrenal)  Pulmonary dysfunction  Renal dysfunction  Metabolic insufficiency  Pre-existing comorbid conditions particularly in elderly  Hepatic dysfunction |  |
| Medication review | Alcohol / recreational drug use  Opioids  Antihistamines  Antidepressants |  |
| Make referrals to appropriate specialist / health professional | Shared responsibility in collaboration with patient. E.g. cardiologist, endocrinologist, mental health professional, rehab / physiotherapy |  |
| Conduct physical examination | Gait, Posture  Range of motion  Eyes, Oral assessment  Muscle wasting  Tachycardia  Shortness of breath |  |
| **Laboratory evaluations** | If screened positive for fatigue – e.g.  Complete blood count  Fluid electrolytes (Na, Ca, K, Mg)  Endocrine: TSH  Adrenal, testosterone |  |

| **3. Treatment recommendations - Adults with CRF, any stage of illness** | |  |
| --- | --- | --- |
| Treat contributing factors | **Algorithm:**  Treat contributing factors.  Optimise sleep quality |  |
| Monitor fatigue | **Algorithm:**  Encourage use of treatment log or diary to monitor fatigue levels & identify peak energy periods |  |
| - **Psychosocial interventions** | |  |
| Psycho-education   - Fatigue education and - Self-management recommendations | **GRADE: Moderate.**  All patients are likely to benefit from routine fatigue education with emphasis on self-care, coping techniques, energy and activity management  Education should be targeted to fatigue with a minimum of 3 sessions  **GRADE: Strong**  Cancer services should promote access to multi-component, group psycho-education programs targeted to self-management of fatigue |  |
| Self-management   - Energy conservation and activity management | **Algorithm:**  Prioritize and pace activities  Delegate  Balance rest and activities  Encourage use of treatment log or diary to plan activities |  |
| Cognitive behavioural therapy (CBT) | **GRADE: Strong**  Referral to experts trained in CBT specifically targeted to fatigue should be offered to survivors with chronic cancer fatigue |  |
| Mindfulness therapies | **NCCN: Level 2A**  Preliminary evidence that mindfulness based interventions may improve fatigue |  |
| Attention-restoring activities | **Consensus**  Reading, games, music, gardening, experience in nature may distract from fatigue |  |
| - **Lifestyle interventions** | |  |
| Physical activity (PA) / exercise | **GRADE: Strong** – benefits outweigh harms.  Counsel all patients as is safe to engage in moderate-intensity PA for ≥30 mins on five or more days of the week or vigorous PT for ≥20 mins three or more days per week.  Progressive resistance training at least 3 days per week in combination with PA for most patients.  Lack of consensus on optimal exercise dose  Evidence in post-treatment mostly established for breast, CRC and prostate.  All types of PA at lower levels of intensity may contribute to decreasing fatigue post-treatment |  |
| Referrals to other practitioners | **NCCN: Category 2A**  A referral to a specialist in rehabilitation should be considered for obese or inactive patients and those requiring tailored regimes (neuropathy, lymphoedema) |  |
| Yoga | **NCCN: Category 2A**  Preliminary evidence that yoga may improve CRF post-treatment |  |
| Nutrition | Treat nutrition deficit (contributing factor) |  |
| Acupuncture, acupressure | **GRADE: Weak**  Insufficient evidence [See (5) for 2018 update] |  |

| - **Pharmacological and herbal medicine** | |  |
| --- | --- | --- |
| Pharmacological management   - Psycho-stimulants - Methylphenidate - Modafinil - Antidepressants | **GRADE: Strong**  Harms may outweigh benefits. Evidence is insufficient to recommend pharmacological agents for fatigue at any stage of disease.  *Other guidelines advise steroids (e.g. dexamethasone) may offer short term relief in advanced / end stage disease (1)* |  |
| Erythropoiesis-stimulating agents (ESA) | *N.B. Not included in CAPO guideline however when used for fatigue (in the absence of anaemia), is associated with increased risk of decreased survival and thrombo-embolus(6). Other CRF guidelines state ‘use with caution’ (1, 7)* |  |
| Nutritional supplements and herbal remedies | **GRADE: Strong**  All herbal products should be used with caution and discussed with oncology team  Insufficient evidence for effectiveness of Paullinia Cupana (guarana), ginseng and other herbal medicines in reducing fatigue.  **GRADE: Weak** – Likely to be effective (ONS)^1^  No evidence for CoQ10 for reducing fatigue |  |

^1^LTBE = likely to be effective = *Interventions for which effectiveness has been demonstrated from a single rigorously conducted controlled trial, consistent supportive evidence from well-designed controlled trials using small samples, or guidelines developed from evidence and supported by expert opinion.* ([https://www.ons.org/practice-resources/pep accessed 11 June 2015](https://www.ons.org/practice-resources/pep%20accessed%2011%20June%202015))

References

1. National Comprehensive Cancer Network. NCCN Clinical Practice Guidelines in Oncology: Cancer-related Fatigue Version 1.2018 2018. Available from: <https://www.nccn.org/professionals/physician_gls/pdf/fatigue.pdf>.

2. Howell D, Keshavarz H, Broadfield L, Hack T, Hamel M, Harth T, et al. A pan Canadian practice guideline for screening, assessment, and management of cancer-related fatigue in adults Version 2-2015 Toronto: Canadian Association of Psychosocial Oncology; 2015. Available from: <https://www.capo.ca/wp-content/uploads/2010/10/CRF_Final.-20150521.pdf>.

3. The ADAPTE Collaboration. The ADAPTE Process: Resource toolkit for guideline adaptation Version 2.0 Scotland2009. Available from: <http://www.g-i-n.net>.

4. Guyatt GH, Oxman AD, Vist GE, Kunz R, Falck-Ytter Y, Alonso-Coello P, et al. GRADE: an emerging consensus on rating quality of evidence and strength of recommendations. BMJ. 2008;336(7650):924-6.

5. Zhang Y, Lin L, Li H, Hu Y, Tian L. Effects of acupuncture on cancer-related fatigue: a meta-analysis. Support Care Cancer. 2018;26(2):415-25.

6. Minton O, Richardson A, Sharpe M, Hotopf M, Stone PC. Drug therapy for the management of cancer-related fatigue. Cochrane Database of Systematic Reviews. 2010;2010(7):CD006704.

7. Mitchell SA, Hoffman AJ, Clark JC, DeGennaro RM, Poirier P, Robinson CB, et al. Putting evidence into practice: an update of evidence-based interventions for cancer-related fatigue during and following treatment. Clin J Oncol Nurs. 2014;18 Suppl:38-58.
