# Supplementary material for: Identifying strategies for implementing a clinical guideline for cancer-related fatigue: a qualitative study
Source: BMC Health Serv Res. 2023 Apr 24;23:395. doi: 10.1186/s12913-023-09377-9 (PMC10127293; doi:10.1186/s12913-023-09377-9)
Supplement: Supplementary file 2 — Additional file 2. [file 12913_2023_9377_MOESM2_ESM.docx]

| **CAPO guideline recommendations [1]** | **Current practice at Cancer Centre** |
| --- | --- |
| 1. **Fatigue Screening** | |
| - 1. *Screen for presence of cancer fatigue: At diagnosis or first intake visit with a health provider; Start of/throughout treatment at specific interval (e.g. Start, midpoint, and end) or with advanced disease; post-treatment follow-up visits; as clinically indicated-changes in disease status or treatment.* | **Routine fatigue or symptom screening**  Variable time points or intervals of screening.   - Day chemotherapy – symptoms, before new cycle (nurse) - Radiotherapy – symptoms weekly (nurse) - Outpatient encounters – symptoms (nurse, doctor, allied health) - Palliative care – every encounter (nurse, doctor) - Inpatients – symptoms every shift (nurse), nutrition status on admission / weekly (nutrition assistant), fatigue (OT/PT) - Surgical inpatients – pre-admission questionnaire (self-completed) - OT / PT outpatients – function, symptoms, fatigue (allied health assistant by phone) |
| - 1. *Screen for cancer fatigue severity using a valid quantitative measure with established cut-offs for severity, or a semi-quantitative measure*. | Fatigue ratings included qualitative (e.g. ‘somewhat’), 0-10 NRS, CTCAE [2], Distress Thermometer [3], PCOC Assessment [4], BFI [5] |
| 1. **Fatigue assessment** | |
| - 1. *Complete a focused assessment if screened positive for fatigue (Score >2 on a 0-10 NRS) to determine onset, pattern and duration; Extent of interference with work, activity, mood; Contributing factors (physical activity, other symptoms-pain, insomnia, depression); Pre-existing co-morbid conditions; person’s beliefs, values, and knowledge about fatigue.*   2. *Complete a comprehensive assessment including laboratory tests if screened positive for fatigue (Score >2 on a 0-10 NRS) to determine/treat medical causes* | Focused fatigue assessment and management of contributing factors occurred regularly in OT and the multidisciplinary allied health ‘Optimisation’ clinic [6], including fatigue measurement using the BFI [5].  Doctors and palliative care nurses investigated related symptoms based on personal algorithms or PCOC assessment [4].  Ward, radiotherapy, chemotherapy and specialist nurses used standard patient health observations and assessments within their scope of practice. |
| - 1. *As a shared responsibility, the interdisciplinary team in collaboration with the patient should discuss any need for referral to specialists for further evaluation*. | Most HPs un aware of specialist referral options for fatigue management.  OTs had expertise in energy management.  Referrals to OT and optimisation clinic were made by some nurse specialists. |
| 1. **Psycho-educational interventions** | |
| - 1. *All patients are likely to benefit from routine patient education about fatigue that emphasizes self-care, coping techniques, energy, and activity management* | Psycho-education for fatigue was inconsistent, often ad hoc in relation to a general HP enquiry about what was happening for the patient. Advice was mostly general.  HPs considered existing printed CRF education resources useful but too long or complex for many people with fatigue.  Day chemotherapy nurses conducted routine education sessions before a new treatment started. This included a pack with a wide range of printed information (not including fatigue), that was noted as excessive for patients to engage with at times.  Some staff e.g. nurses, PTs and OTs provided individual energy management education as part of usual care. |
| - 1. *Cancer services should promote access to multi-component, group psycho-education programs targeted to self-management of fatigue for patients and survivors* | A monthly OT face-to-face group fatigue education session was usually attended by a few people with mild to moderate fatigue. Patients with moderate to severe fatigue were often too tired to attend, and individual fatigue education was considered more appropriate.  A YouTube version on the hospital website was difficult to find. |
| - 1. *Referral to experts or fatigue clinics that are trained in cognitive behavioural therapy (CBT) specifically targeted to fatigue should be offered to patients and those with chronic cancer fatigue as survivors*. | The clinical psychology department provided CBT programs for insomnia, anxiety and fear of cancer recurrence but none specifically targeted to cancer fatigue, and none existed in the community. |

| 1. **Physical activity / Exercise** | |
| --- | --- |
| - 1. *Counsel all patients as is safe to engage in moderate-intensity physical activity 55-75% for at least 30 minutes on five or more days of the week, or vigorous-intensity physical activity for at least 20 minutes on three or more days of the week (e.g. fast walking, cycling or swimming).*   2. *All types of physical activity at lower levels of intensity (i.e. walking, yoga) likely will contribute to decreasing fatigue for most patients during active treatment and post-treatment survivorship*   3. *Patients should be advised that there is preliminary evidence that yoga is likely to improve cancer fatigue*. | PTs and exercise physiologists treated inpatients and outpatients individually and in group classes, predominantly for conditioning and safe mobility, not fatigue management.  Exercise was prescribed: never ‘one size fits all’ but rather targeted at a level that could be achieved by the patient.  Home based programs were considered more feasible than community classes.  Recommendation 4.1 was considered inappropriate for people with significant fatigue.  Exercise bikes located in the chemotherapy unit and haematology ward could only be used under supervision, which was seldom available. |
| BFI - Brief Fatigue Inventory; CBT – Cognitive behavioural therapy; CTCAE – Common toxicity criteria for adverse events [2]; NRS – Numeric rating scale; OT – occupational therapy; PCOC – Palliative Care Outcomes Collaboration; PT – Physiotherapy; RT – Radiotherapy | |

**References**

1. **A Pan Canadian Practice Guideline for Screening, Assessment, and Management of Cancer-Related Fatigue in Adults Version 2-2015** [<https://www.capo.ca/>]

2. U.S. Department of Health and Human Services: **Common Terminology Criteria for Adverse Events (CTCAE) Version 5.0**. In*.*: National Institutes of Health; 2017.

3. Abrahams HJG, Gielissen MFM, de Lugt M, Kleijer EFW, de Roos WK, Balk E, Verhagen CAHHVM, Knoop H: **The Distress Thermometer for screening for severe fatigue in newly diagnosed breast and colorectal cancer patients**. *Psychooncology* 2016(26):693-697.

4. Palliative Care Outcomes Collaboration: **Clinical Manual**. In*.* Edited by Clapham S, Holloway A. Wollongong, NSW: University of Wollongong; 2014.

5. Mendoza TR, Wang XS, Cleeland CS, Morrissey M, Johnson B, Wendt JK, Huber SL: **The rapid assessment of fatigue severity in cancer patients - use of the Brief Fatigue Inventory**. *Cancer* 1999, **85**:1186-1196.

6. Ray H, Beaumont A, Loeliger J, Martin A, Marston C, Gough K, Bordia S, Ftanou M, Kiss N: **Implementation of a Multidisciplinary Allied Health Optimisation Clinic for Cancer Patients with Complex Needs**. *Journal of Clinical Medicine* 2020, **9**(8):2431.
